# Paris and Its Drains

**Published:** 1901-06-08

**Authors:** 


### PARIS AND ITS DRAINS.

Now that after much labour and expense Paris has got its drains, it is becoming a question of much moment what it will do with them. In olden days the sewers of Paris were not intended to carry what we mean by sewage. They emptied into the Seine, which twisting about in its course through and around the growing city, would have deposited such sewage refuse almost at its gates had the attempt been made to utilise the drains as a means of disposing of sewage proper. Even the slop-water, which the householders were allowed to empty down them, and the ordinary washings of the streets were together sufficient to make the Seine by no means pleasant, and it was quite out of the question to allow the closets to drain into the sewers until some means were found for utilising, or at least for purifying, the effluent. A couple of years ago, however, after the expenditure of a large amount of money during many years, the outfall works were at last completed, the objection to draining closets into the sewers was removed, and it was hoped that the good Parisians would joyfully accept the facilities put in their way of at least diminishing the somewhat notorious odours of Paris. So far as the Parisians themselves are concerned, we have no doubt they would quite willingly accept any benefit which the good God might offer to them free of charge, even a decent system of house drainage. But the houses of Paris are mostly built on the "flat" system, and the proprietors of these flats by no means appreciated the advantage of having to pay for providing their tenants with proper drains. Hence the business has gone on with miserable slowness, and the splendid sewers provided by the municipality are only very partially made use of. A correspondent of the *Lancet* says that there are 78,000 houses in Paris, and that only 24,000 of these now possess water-closets and drain direct into the sewers. For the remaining 54,000 houses, however, there are still in existence 47,913 cesspools, 21,813 *tinettes filtrantes* (metallic pails which, while retaining the solid matter, allow the liquids to flow to the sewer), and 11,909 wooden tubs or pails which retain both solids and liquids. Those who have not had the delicious experience of living in the unreformed quarters of Paris may wonder how things go on in buildings of many "flats" furnished only with such appliances; even those who have lived there have often been content not to inquire too closely, notwithstanding the warning given by the nose. Still it is as well to know. These cesspools are supposed to be emptied once a year, and the pails removed once a week or oftener, and, of course, under either of these systems, there is no water in the closet and no syphon traps can be applied. A pan can only be cleaned with a brush, and by pouring a little water on it from a hand-jug. If much water is used the landlord soon finds it out, for then he has to empty his cesspool, to doing which he objects greatly on account of the expense. Naturally the whole arrangement is always filthy, and there is nothing to prevent the foul air from the soil-pipe and the cesspool entering the house. Oh, these *odeurs de Paris!* How we wonder that we are still alive! Yet the Parisian house proprietors have actually founded a syndicate for the protection of their interests, in other words to resist the efforts of the municipality to enforce the use of the sewers that have been provided.

